# Metastatic Melanoma with an Unknown Primary Origin Presenting with Postmenopausal Bleeding: A Case Report

**DOI:** 10.3390/reports9030309

**Published:** 2026-09-12

**Authors:** Nulvin Djebbara-Bozo, Nikolaj Warming, Abdullah N. Maarouf, Bence Gabor Szilvasy

**Affiliations:** 1 Department of Breast and Plastic Surgery 1, Aalborg University Hospital, Hospitalsbyen 1, 9260 Gistrup, Denmark; n.warming@rn.dk (N.W.); abdm@rn.dk (A.N.M.); 2 Department of Pathology 2, Aalborg University Hospital, Hospitalsbyen 1, 9260 Gistrup, Denmark; b.szilvasy@rn.dk

**Keywords:** melanoma of unknown primary, endometrial metastasis, metastatic melanoma, postmenopausal bleeding, gynecologic oncology, immunohistochemistry, nivolumab, immunotherapy

## Abstract

**Background and Clinical Significance:** Melanoma is predominantly a cutaneous malignancy, whereas metastatic involvement of the endometrium is exceptionally rare. Melanoma of unknown primary (MUP) accounts for a small portion of such cases. Patients typically present with abnormal uterine bleeding, and diagnosis can be challenging because metastatic melanoma can mimic other uterine malignancies. We report a rare case of MUP presenting with synchronous endometrial and subcutaneous metastases, a combination not previously reported in the literature. **Case Presentation**: A multiparous woman in her seventies presented with persistent postmenopausal bleeding. Transvaginal ultrasonography demonstrated endometrial thickening, and endometrial biopsy revealed metastatic melanoma confirmed by immunohistochemical positivity for S100, SOX10, HMB-45, and Melan-A. Positron emission tomography–computed tomography identified fluorodeoxyglucose uptake in the uterine corpus and a metabolically active subcutaneous lesion in the right anterolateral abdominal wall. Surgical excision of the abdominal lesion confirmed metastatic melanoma. Despite extensive investigations, including dermatological examination, ophthalmological assessment, and imaging studies, no primary tumor was identified. The patient underwent hysterectomy followed by adjuvant nivolumab. At 24-month follow-up, no evidence of disease recurrence was detected. **Conclusions**: Endometrial metastasis of melanoma may occur in the absence of an identifiable primary lesion and should be considered in patients presenting with abnormal uterine bleeding, particularly those with a history of melanoma or unexplained metastatic disease. Because these lesions frequently present as polypoid, nodular, or infiltrative endometrial masses, prompt hysteroscopy and endometrial sampling are essential for diagnosis. This case highlights the importance of comprehensive histopathological evaluation and multidisciplinary management in rare presentations of metastatic melanoma.

## 1. Introduction and Clinical Significance

Melanoma is predominantly a cutaneous malignancy, accounting for approximately 1% of all malignant tumors. Although most melanomas arise from the skin, extracutaneous mucosal melanomas account for approximately 1.3% of all melanoma cases [1,2] and are associated with significantly poorer outcomes than cutaneous melanoma [3,4,5]. Melanoma involving the female genital tract is uncommon and most frequently affects the vulva and vagina, whereas uterine involvement is exceptionally rare [2]. Most cases involving the endometrium represent metastatic disease originating from a known cutaneous melanoma or, less commonly, a mucosal primary lesion [6,7,8,9]. Melanoma of unknown primary (MUP) involving the endometrium appears to be exceptionally rare. In our literature review, no previously reported case could be unequivocally classified as metastatic melanoma of unknown primary involving the endometrium. Similarly, only one case of primary endometrial melanoma has been reported to date [2].

We describe a rare case of MUP presenting with synchronous subcutaneous metastases. To our knowledge, this combination has not previously been reported. The case highlights the importance of considering metastatic melanoma in patients presenting with abnormal uterine bleeding, even when no primary lesion can be identified.

## 2. Case Presentation

### 2.1. Patient Information and Clinical Presentation

A multiparous woman in her seventies was referred by her general practitioner to the Department of Gynecology and Obstetrics because of persistent daily postmenopausal bleeding for three weeks. She had been postmenopausal for more than two decades and reported no associated pelvic pain, weight loss, or constitutional symptoms. Her medical history included bilateral salpingo-oophorectomy performed one year earlier for a benign ovarian cyst. She had no personal or family history of cutaneous melanoma or other melanoma. However, she had a red-haired phenotype and Fitzpatrick skin type I, which is a recognized risk factor for melanoma. Upon gynecological examination, no abnormalities were identified in the vulva, vagina, or cervix. Transvaginal ultrasonography demonstrated irregular endometrial thickening, prompting further evaluation. Sonohysterography subsequently identified two endometrial polyps measuring 8 × 4 mm and 10 × 3 mm, respectively. Pipelle biopsy was obtained to exclude endometrial malignancy.

### 2.2. Diagnostic Work-Up

Initial histopathological examination of the endometrial biopsy revealed a poorly differentiated malignant neoplasm suspicious for uterine sarcoma. Given the unusual morphology, the specimen was referred to a tertiary pathology center for expert review. Additional immunohistochemical analysis demonstrated diffuse positivity for S100, SOX10, HMB-45, and Melan-A, findings consistent with melanoma. At this stage, it remained unclear whether the lesion represented a primary endometrial melanoma or metastatic disease. Routine laboratory investigations, including complete blood count, renal function, and liver function tests, were within normal limits. No tumor markers were obtained. To determine disease extent and identify a possible primary lesion, a whole-body positron emission tomography–computed tomography (PET-CT) scan was performed. The examination demonstrated intense fluorodeoxyglucose (FDG) uptake within the uterine corpus corresponding to the biopsy-proven lesion. In addition, several bilateral pulmonary nodules measuring approximately 5 mm demonstrated faint FDG uptake and were scheduled for radiological surveillance. A metabolically active subcutaneous lesion was also identified in the right anterolateral abdominal wall. The case was subsequently discussed at a multidisciplinary tumor board involving gynecologists, pathologists, oncologists, nuclear medicine physicians, and plastic surgeons. Given the rarity of the diagnosis and the uncertainty regarding the primary site, comprehensive staging investigations and surgical management were recommended.

### 2.3. Histopathological Findings

**Figure 1:**
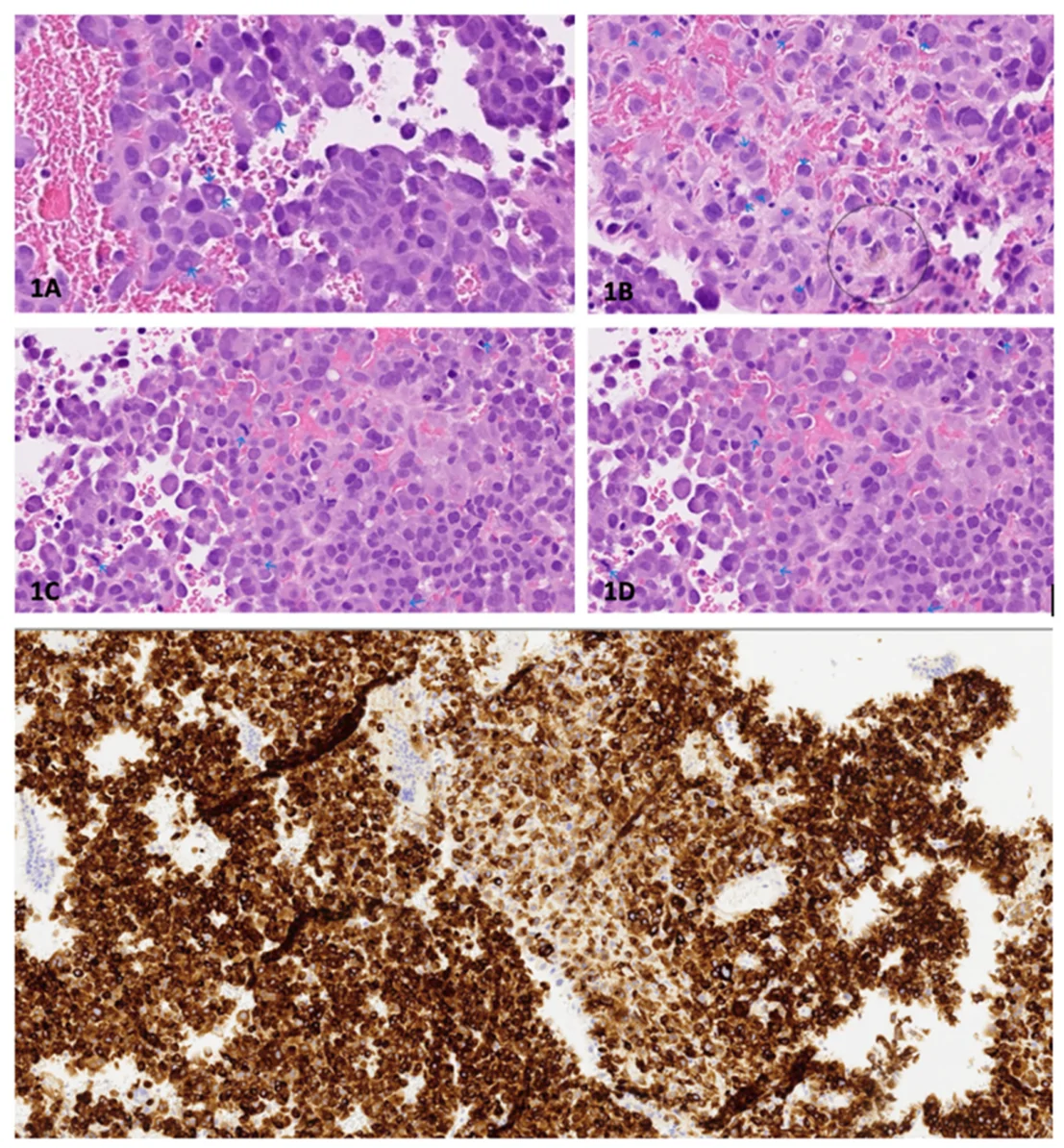
Endometrial biopsy. (**1A**) The tumor cells are relatively large with abundant eosinophilic cytoplasm. Prominent cytoplasmatic inclusions are present in some nuclei (arrows). (**1B**) HE 400×: enlarged nuclei with prominent nucleoli (arrows). Inconspicuous brown pigment is present in a cell (circle). (**1C**) HE 400×: mitotic, as are evident (arrows). (**1D**) HE 400×: the tumor cells are strongly immunoreactive for Melan-A (MART-1).

Microscopic examination of the endometrial biopsy demonstrated fragmented benign atrophic endometrium admixed with sheets of malignant epithelioid cells (see Figure 1). The tumor cells were moderately enlarged and exhibited abundant eosinophilic cytoplasm, enlarged round-to-oval nuclei, prominent nucleoli, and mild pleomorphism. Cytoplasmic inclusions were observed, and scattered binucleated and multinucleated tumor cells were present. Mitotic figures were readily identifiable. Rare tumor cells contained a fine brown cytoplasmic pigment suggestive of melanin. Immunohistochemical analysis demonstrated strong positivity for S100, SOX10, HMB-45, Melan-A (MART-1), CD10, Cyclin D1, and focal WT-1 expression. The tumor cells were negative for PAX8, pan-cytokeratin, CD34, myogenin, CD45, estrogen receptor, progesterone receptor, desmin, ASMA, and p16. BRAF/NRAS mutation analysis identified NRAS mutation.

Based on the combined morphological and immunophenotypic findings, the lesion was diagnosed as melanoma.

**Figure 2:**
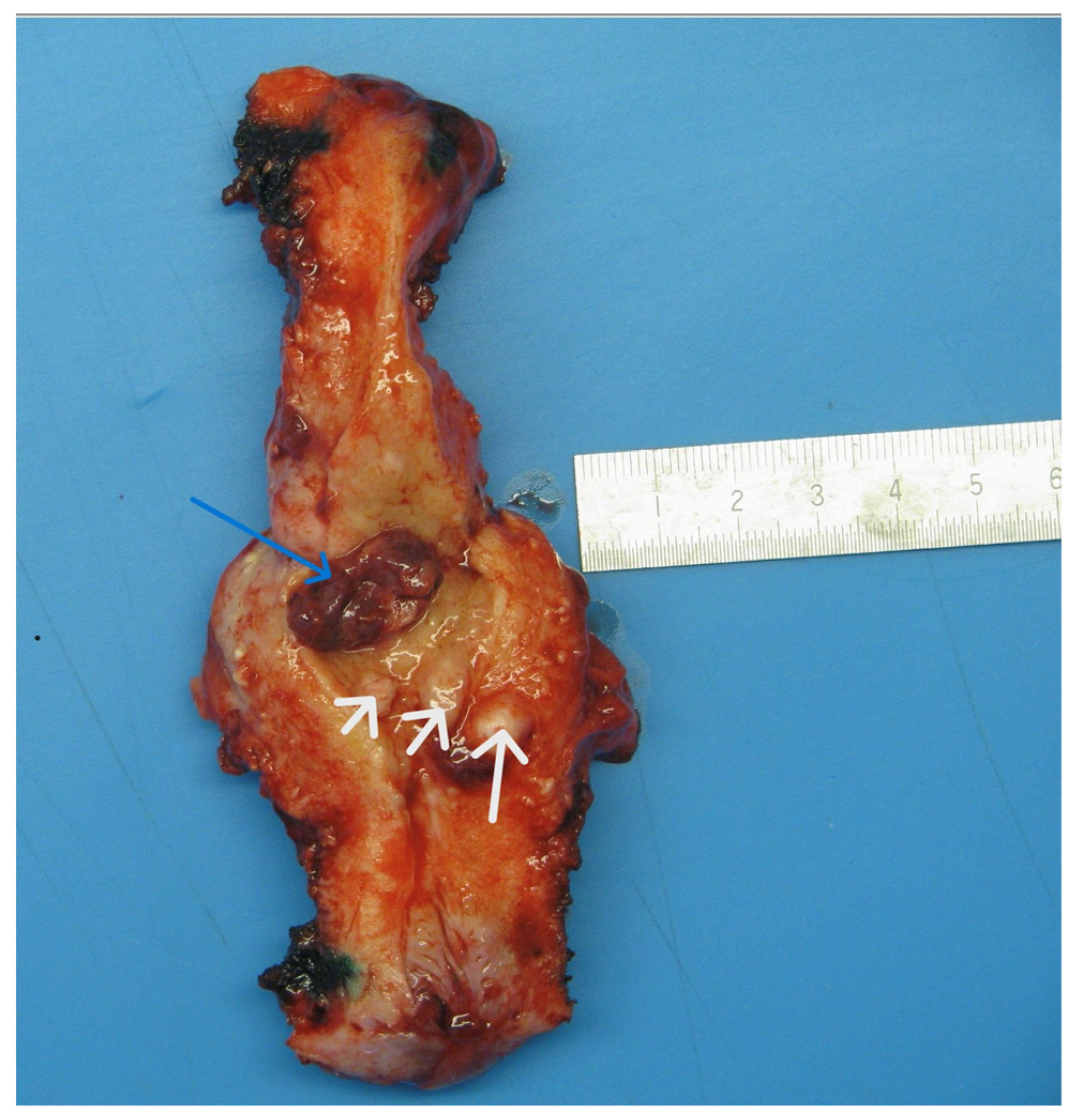
Hysterectomy specimen. Reddish brown polypoid mass in uterine fundus (blue arrow). Uterus was normal in size and shape. No cervical or serosal lesions were observed. After opening the uterine cavity, a polypoid mass was seen in the fundus region. The whitish round lesions represent submucous polypoid and intramural leiomyomas (white arrows).

**Figure 3:**
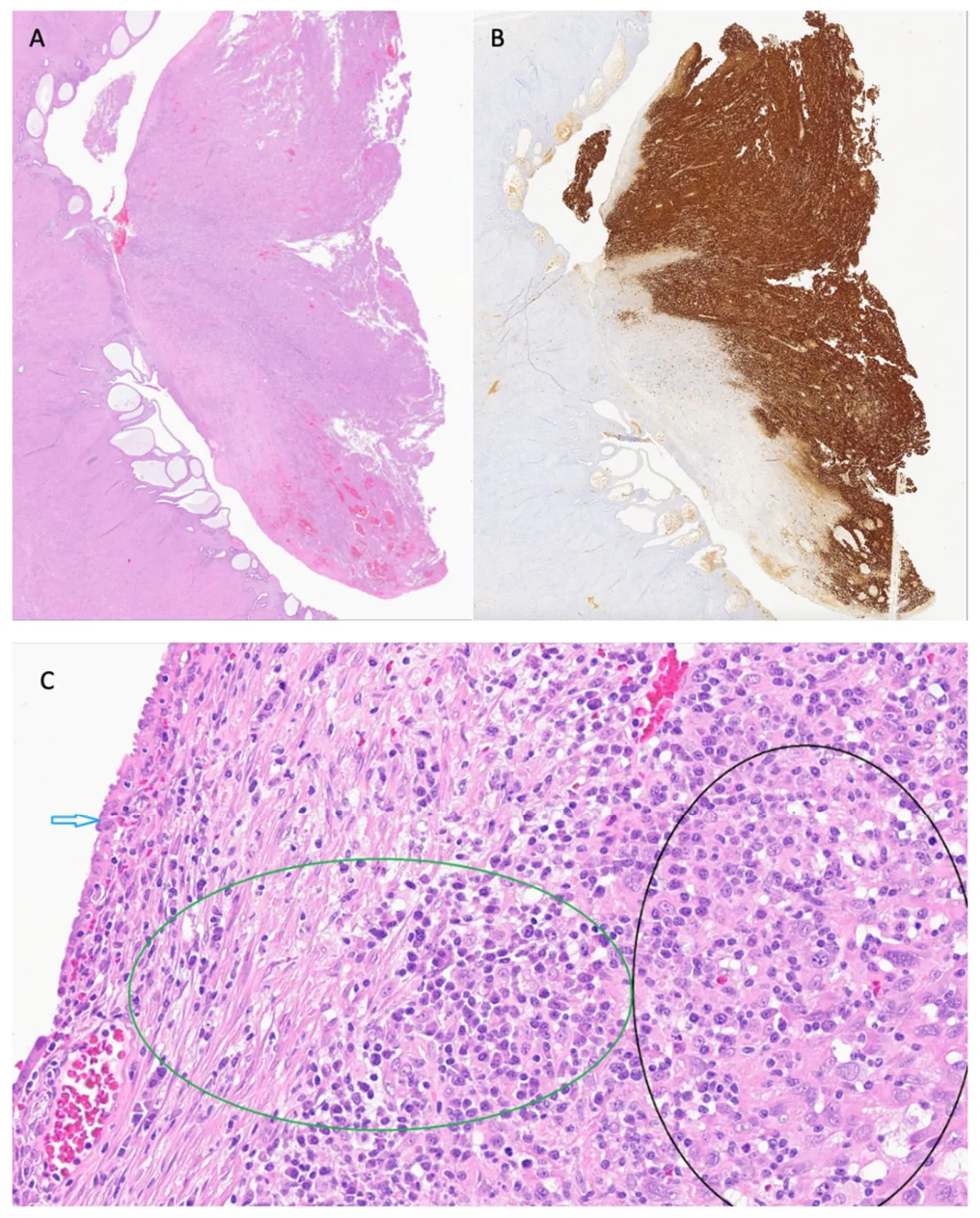
(**A**) Whole mount sagittal section of uterine fundus; S100, 50×. Endometrium is atrophic. (**B**) S100 immunostain highlights tumor growth confined to polyp. (**C**) Benign-looking surface epithelium (blue arrow). Plasma cell-rich inflammatory infiltrate in subepithelial stroma (green circle). Malignant cells in deep stroma (black circle); HE, 400×.

After hysterectomy, the uterus was evaluated macroscopically and microscopically (see Figure 2 and Figure 3).

### 2.4. Treatment

**Figure 4:**
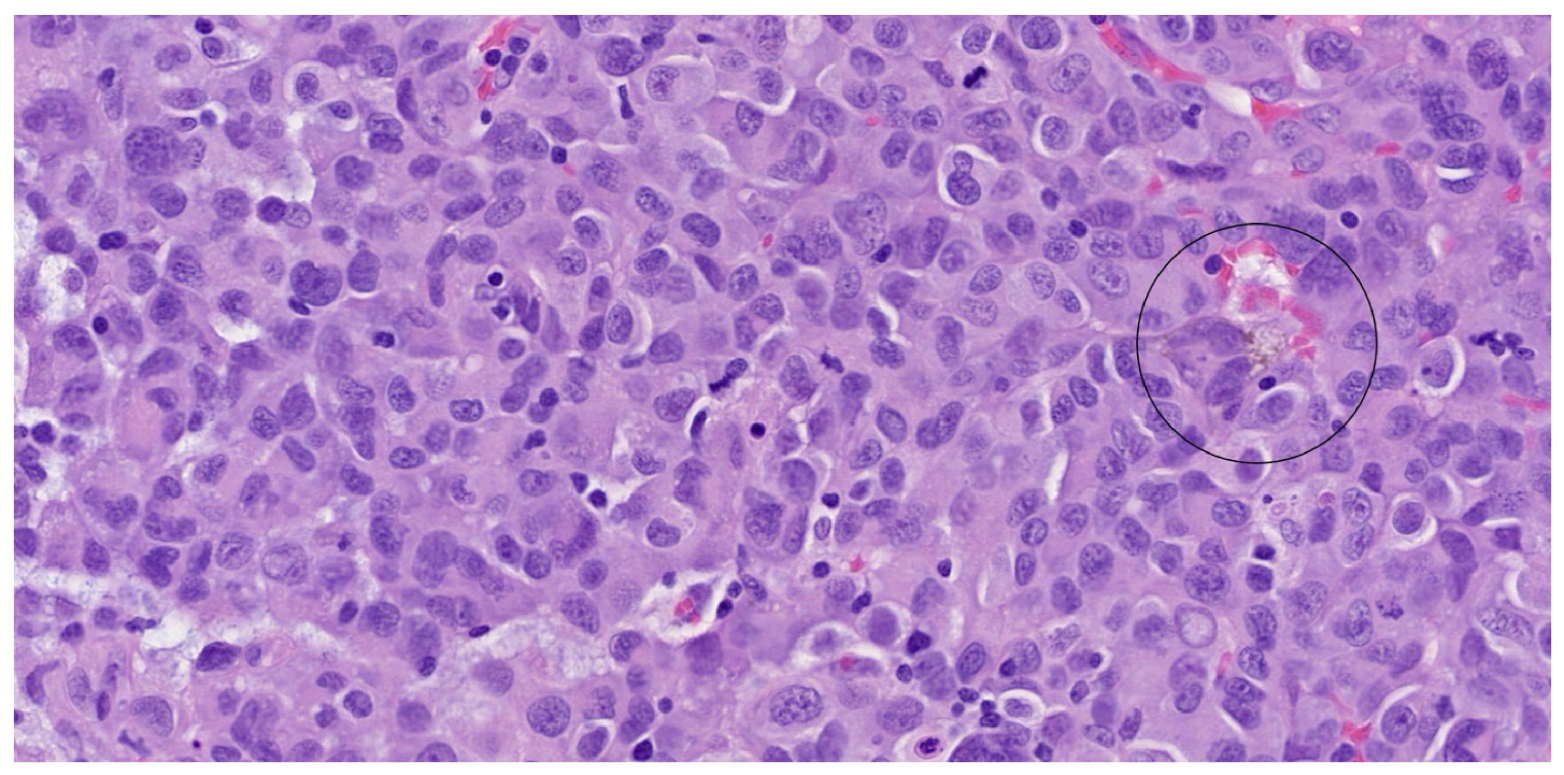
The excision biopsy from the abdominal wall consisted of mainly fatty tissue measuring 20 mm with a centrally placed 13 mm lesion. Microscopically, the lesion was similar to the endometrial biopsy and hysterectomy specimen. The case was signed out as metastatic melanoma of probable cutaneous origin. Vague pigmentation (circle), HE 400×.

Dermatological examinations were performed by both a dermatologist and a plastic surgeon, including examination of the scalp, nails, and acral surfaces. No suspicious pigmented lesions or other lesions requiring further investigation were identified. A dedicated upper aerodigestive mucosal examination and anorectal examination were not performed. Ophthalmological assessment likewise failed to identify an ocular primary melanoma. Because PET-CT demonstrated a metabolically active lesion within the abdominal wall, the lesion was localized preoperatively using ultrasound-guided Magseed placement and subsequently excised under local anesthesia. Intraoperatively, the lesion appeared as a firm subcutaneous mass with a bluish discoloration. Histopathological examination confirmed metastatic melanoma involving adipose tissue, with morphological features identical to those observed in the endometrial biopsy specimen (Figure 4).

Following renewed multidisciplinary review, the patient underwent hysterectomy. Macroscopic examination of the resected uterus revealed a reddish-brown polypoid lesion located in the uterine fundus and some well-circumscribed whitish submucous and intramural tumors, consistent with leiomyoma. The uterus was otherwise normal in size and configuration, and no cervical or serosal abnormalities were observed. Histological examination of the hysterectomy specimen showed normal cervix, atrophic endometrium, submucous and intramural leiomyomas, and normal serosa. Slides from the in toto sampled brownish polypoid lesion showed identical histology with the biopsy, i.e., malignant melanoma. No benign melanocytic lesion or tumor cells mimicking intraepithelial growth of skin melanoma were found. The tumor growth was confined to the polyp and there were no signs of vascular or myometrial invasion. All surgical margins were negative for melanoma. The histological slides from the previous bilateral salpingo-oophorectomy were reviewed, confirming the original benign diagnosis, with no evidence of melanocytic lesions or metastatic melanoma. Following review of the complete clinical and histopathological findings, the case was re-assessed by the multidisciplinary tumor board. The classification as metastatic melanoma of unknown primary (MUP) was based on several considerations. First, two anatomically distant lesions, located in the endometrium and the subcutaneous tissue of the abdominal wall, showed identical histopathological features. Second, neither lesion was associated with an identifiable precursor lesion or showed evidence of locoregional spread. In particular, no in situ or junctional melanocytic component was identified in the endometrial epithelium. Third, primary melanoma of the endometrium is exceedingly rare, whereas MUP is a well-recognized clinical entity. Finally, despite comprehensive clinical evaluation, no primary melanoma was identified. Taken together, these findings favored metastatic melanoma of unknown primary rather than primary endometrial melanoma, and the patient was classified and treated accordingly. Following surgery and multidisciplinary reassessment, adjuvant treatment with nivolumab was initiated five weeks postoperatively. Nivolumab was administered every four weeks according to the institutional oncology protocol, with a planned treatment duration of one year. The patient completed 13 cycles. The exact dose was not available in the medical records. Reported adverse events during treatment included anemia, fatigue, dyspnea, and weight gain. Despite these adverse events, the patient completed the planned treatment course.

### 2.5. Outcome and Follow-Up

The postoperative course was uncomplicated. Surveillance with PET-CT imaging was scheduled every three months. At one-year follow-up, the patient had resumed normal daily activities and remained clinically well. Follow-up imaging demonstrated no evidence of local recurrence, new metastatic disease, or progression of the previously identified pulmonary nodules. See full timeline in Table 1.

## 3. Discussion

To contextualize the present case, a focused systematic literature search was conducted with the assistance of a medical librarian. PubMed, Scopus, and Google Scholar were searched for published case reports and case series of melanoma involving the uterus, including both primary uterine melanoma and metastatic melanoma involving specific uterine compartments. Reference lists of relevant articles were additionally screened to identify further eligible reports. The search identified multiple previously reported cases of metastatic melanoma involving the endometrium (Supplementary Table S1), the majority representing metastatic disease from a known primary melanoma. No previously reported case could be unequivocally classified as metastatic melanoma of unknown primary. One previously reported case described malignant melanoma involving the endometrium and myometrium in the absence of an identifiable primary lesion; however, the authors did not definitively classify the lesion as primary or metastatic [10]. The available cases, including the primary site, uterine compartment involved, other metastatic sites, treatment, and outcome, are summarized in Supplementary Table S1. The present case is particularly noteworthy because it involved synchronous endometrial and subcutaneous metastases without identification of a primary lesion—a combination that, to our knowledge, has not previously been reported.

The rarity of endometrial involvement contributes to the diagnostic delay. Consistent with previous reports, our patient presented with persistent postmenopausal bleeding, which appears to be the most common clinical manifestation of melanoma involving the endometrium [11,12,13,14,15]. Because abnormal uterine bleeding is a common symptom with a broad differential diagnosis, metastatic melanoma may not initially be considered. However, our findings support previous observations that metastatic melanoma should be included among the rare differential diagnoses in patients presenting with unexplained abnormal uterine bleeding, particularly in individuals with a history of melanoma or unexplained metastatic disease. The diagnosis of endometrial melanoma relies heavily on histopathological examination and immunohistochemical characterization. Metastatic melanoma may mimic poorly differentiated carcinoma, sarcoma, or other malignant uterine neoplasms, making immunohistochemistry essential. S100 is considered the most sensitive marker for melanoma, whereas HMB-45 and Melan-A are more specific melanocytic markers. SOX10 has also emerged as a highly sensitive marker for melanocytic differentiation. In the present case, strong positivity for S100, SOX10, HMB-45, and Melan-A supported the diagnosis of melanoma and enabled distinction from other poorly differentiated uterine malignancies. The initial suspicion of sarcoma further illustrates the diagnostic complexity of these rare lesions. Carcinosarcoma with melanocytic differentiation should also be considered in differential diagnosis. In the present case, however, extensive histological examination of the specimen revealed no epithelial or sarcomatous component. Macroscopically, melanoma involving the endometrium has been described as polypoid, nodular, or diffusely infiltrative, frequently demonstrating varying degrees of pigmentation [16,17,18]. Among previously reported cases, polypoid lesions appear to be a relatively common presentation. Similarly, the uterine lesion in our patient appeared as a reddish-brown polypoid mass located in the fundus, emphasizing the importance of considering melanoma in the differential diagnosis of unusual pigmented or polypoid endometrial lesions. Despite extensive investigations, including comprehensive dermatological examination, imaging studies, and multidisciplinary review, no primary lesion was identified in our patient. An important differential diagnosis was primary endometrial melanoma. However, the absence of an in situ or junctional melanocytic component in the endometrium, together with the presence of a histologically identical synchronous subcutaneous lesion at a distant anatomical site and the absence of regional spread, favored metastatic disease. An additional differential diagnosis was primary cutaneous or dermal melanoma, including melanoma with an intraepidermal component obscured by regression or ulceration, or melanoma arising from a nevus. However, the subcutaneous location, absence of an associated nevus, and histological similarity to the endometrial lesion favored metastatic disease. Nevertheless, an occult or completely regressed cutaneous primary cannot be excluded.

Melanoma of unknown primary origin is thought to account for approximately 2–6% of all melanoma cases and may result from complete regression of the primary lesion or from malignant transformation of ectopic melanocytic cells. Although the exact mechanism remains uncertain, the absence of an identifiable primary tumor should not exclude the diagnosis of metastatic melanoma when clinicopathological findings are otherwise consistent. Management of endometrial metastatic melanoma remains individualized because of the rarity of the condition and the absence of standardized treatment guidelines. Surgical resection remains the cornerstone of treatment when the disease is considered resectable and confined to a limited number of sites. However, melanoma is characterized by aggressive biological behavior and a propensity for both lymphatic and hematogenous dissemination, necessitating careful staging and multidisciplinary management. In recent years, immune checkpoint inhibitors have significantly improved outcomes in advanced melanoma. In our case, complete surgical excision of both metastatic lesions followed by adjuvant nivolumab therapy resulted in no evidence of recurrent disease at 24-month follow-up. Although metastatic melanoma involving the endometrium remains exceedingly uncommon, the increasing number of reported cases suggests that this diagnosis may be underrecognized. Improved pathological techniques, widespread use of immunohistochemistry, and advanced imaging modalities are likely contributing to increased detection. Continued reporting of similar cases is therefore important to improve understanding of disease behavior, optimize diagnostic strategies, and guide future management recommendations.

## 4. Conclusions

Endometrial metastasis from melanoma of unknown primary origin is an exceptionally rare diagnosis, and may present with postmenopausal bleeding as the initial symptom. This case demonstrates that metastatic melanoma should be considered in patients presenting with abnormal uterine bleeding, particularly when histopathological findings are atypical or when metastatic disease is suspected. Accurate diagnosis requires comprehensive histopathological evaluation and immunohistochemical characterization. Early recognition and multidisciplinary management are essential to optimize treatment and patient outcomes.

Key lessons from this case include the following:

- Endometrial metastasis may occur in the absence of an identifiable primary melanoma.
- Abnormal uterine bleeding in patients with a history of melanoma or unexplained metastatic disease should prompt hysteroscopy and endometrial sampling.
- Melanoma involving the endometrium commonly presents as a polypoid, nodular, or infiltrative lesion and requires immunohistochemical confirmation for definitive diagnosis.

## Figures and Tables

**Table 1:** Timeline of the clinical course according to CARE guidelines.

Time	Clinical Event	Findings/Management
1 year before presentation	Previous surgery	Bilateral salpingo-oophorectomy was performed for a benign ovarian cyst. The histological slides were subsequently reviewed and confirmed the original benign diagnosis, with no evidence of a melanocytic lesion or metastatic melanoma.
Presentation	Postmenopausal bleeding	The patient presented with persistent daily postmenopausal bleeding for three weeks. Transvaginal ultrasonography demonstrated irregular endometrial thickening.
Initial diagnostic work-up	Sonohysterography and endometrial sampling	Sonohysterography identified two endometrial polypoid lesions measuring 8 × 4 mm and 10 × 3 mm. Endometrial sampling was performed using a Pipelle biopsy.
Pathology review	Histopathological diagnosis	Initial examination suggested a poorly differentiated malignant neoplasm suspicious for uterine sarcoma. Expert pathology review and immunohistochemistry (positive for S100, SOX10, HMB-45, and Melan-A) established the diagnosis of melanoma.
Staging	PET-CT and search for primary melanoma	PET-CT demonstrated FDG uptake in the uterine corpus, a metabolically active subcutaneous lesion in the right anterolateral abdominal wall, and small bilateral pulmonary nodules. Comprehensive dermatological and ophthalmological examinations did not identify a primary melanoma.
Following staging	Excision of abdominal wall lesion	The subcutaneous abdominal wall lesion was localized using ultrasound-guided Magseed placement and surgically excised. Histopathological examination confirmed melanoma with morphological features identical to those of the endometrial lesion.
Following multidisciplinary review	Hysterectomy	Hysterectomy was performed. Histopathological examination demonstrated melanoma confined to an endometrial polyp, without involvement of the surrounding endometrium, myometrial invasion, or lymphovascular invasion. No in situ or junctional melanocytic component was identified. Surgical margins were negative. The additional polypoid lesions were benign leiomyomas.
5 weeks postoperatively	Adjuvant nivolumab initiated	Nivolumab was initiated and administered every four weeks, with a planned treatment duration of one year.
During the following year	Adjuvant treatment	The patient completed 13 cycles of nivolumab. Reported adverse events included anemia, fatigue, dyspnea, and weight gain; treatment was completed as planned.
24 months after surgery	Latest follow-up	No evidence of disease recurrence was detected.

## Data Availability

The original contributions presented in this study are included in the article. Further inquiries can be directed to the corresponding author.

## Supplementary Materials

The following supporting information can be downloaded at: https://www.mdpi.com/article/10.3390/reports9030309/s1, Table S1: Previously reported cases of metastatic melanoma involving the endometrium. References [19,20,21,22,23,24,25,26,27,28,29,30,31,32,33,34,35] are cited in the supplementary materials.

## Abbreviations

The following abbreviations are used in this manuscript:

MUP	Melanoma of unknown primary
