# Supplementary material for: Metastatic Melanoma with an Unknown Primary Origin Presenting with Postmenopausal Bleeding: A Case Report
Source: Reports (MDPI). 2026 Sep 12;9(3):309. doi: 10.3390/reports9030309 (PMC13611414; doi:10.3390/reports9030309)
Supplement: Supplementary file 1 [file reports-09-00309-s001.zip › reports-4486655-supplementary.pdf]

Table S1. Previously reported cases of metastatic melanoma involving the endometrium										
**Number**	**Author, year**	**Age**	**Presenting symptom**	**Primary site**	**Primary known /unknown**	**Primary vs. metastatic**	**Precise uterine compartment involved**	**Other metastatic sites**	**Treatment**	Outcome
1 (1)	Weber, 1971	26	Abnormal uterine bleeding	Cutaneous	Known	Metastatic	Endometrium	None	Uterine extirpation with one ovary	Not reported
2 (2)	Casey et al., 1974	31	Postpartum irregular vaginal bleeding	Cutaneous	Known	Metastatic	Endometrium*	Brain, lungs, liver, adrenals, heart, mesentery, pleura, peritoneum	Hysterectomy + bilateral salpingo-oophorectomy and postoperative chemotherapy	Died around 1 month after diagnosis
3 (3)	Takeda et al.,1978	74	Abnormal vaginal bleeding	Cutaneous	Known	Metastatic	Endometrium;	None	Endometrial curettage + excision of primary skin lesion	Died 5 months later from myocardial infarct
4 (4)	Wood, 1978	61	Postmenopausal brownish vaginal discharge	Cutanous	Known	Metastatic	Endometrium	Bone metastasis in the right superior pubic ramus	Curettage followed by total hysterectomy	At least 4 months after hysterectomy diagnose of uterine metastasis
5 (5)	Scholz et al., 1980	75	Postmenopausal bleeding	Cutaneous	Known	Metastatic	Endometrium	Brain, thyroid, lungs, heart, kidneys, bladder, right ovary, lymph nodes	Fractional curettage	Died 2 months later
6 (6)	Bauer et al., 1984	73	Postmenopausal bleeding	Cutaneous	Known	Metastatic	Endometrium	None	Pelvic radiotherapy + brachytherapy, followed by total hysterectomy and bilateral salpingo-oophorectomy	Alive with no clinical evidence of disease at 2,5 years follow-up
7 (7)	Nagy et al., 1988	38	Uterine bleeding	Cutaneous	Known	Metastatic	Endometrium	†	†	†
8 (8)	Pommerenke et al., 1989	77	Postmenopausal bleeding	Cutaneous	Known	Metastatic	Endometrium	†	†	†
9 (9)	Glaubitz et al., 1992	65	Postmenopausal bleeding	Cutaneous	Known	Metastatic	Endometrium	Lymph nodes, orbit, subcutaneous tissue, brain, stomach, small intestine,	Systemic chemotherapy	Died 10 months after diagnosis

								adrenal glands		
10 (10)	Luxman et al., 1997	60	Vulval lesion, 6 months later postmenopausal bleeding	Vulva	Known	Metastatic	Endometrium	None	Hysterectomy with bilateral salpingo-oophorectomy and pelvic lymph node sampling. Chemo with bilateral salpingo-oophorectomy and pelvic lymph node sampling. Chemotherapy	Disease free at 6 months follow up
11 (11)	Suenaga et al., 1999	75	Genital bleeding	Vagina	Known	Metastatic	Endometrium	†	†	†
12 (12)	Heinig et al., 2001	60	Atypical vaginal bleeding	Cutaneous	Known	Metastatic	Endometrium	Right axillary lymph nodes, right breast, right forearm, and lungs	Excision and radiotherapy followed by chemotherapy. Fractional curettage for the endometrial metastasis	Died 6 months after curettage
13 (13)	Berker et al., 2004	39	Abnormal uterine bleeding	Cutaneous	Known	Metastatic	Endometrium; polypoid lesion in endometrial cavity	None	Total abdominal hysterectomy with bilateral salpingo-oophorectomy and pelvic lymph node sampling	Alive with no clinical evidence of disease at 6-month follow-up
14 (14)	Varras et al., 2004	78	Lower abdominal pain, fever, and constipation	Not identified	Unknown	Uncertain/not established	Endometrium*	None	Total hysterectomy with bilateral salpingo-oophorectomy	Uneventful postoperative recovery; close follow-up recommended (subsequent outcome not reported)
15 (15)	Di Tommaso et al., 2005	47	Uterine bleeding	Cutaneous	Known	Metastatic	Endometrium	None	Hysteroscopic polypectomy, followed by total hysterectomy with bilateral salpingo-oophorectomy	Alive and well without evidence of disease at 18-month follow-up

16 (16)	Giordano et al., 2006	44	Abnormal uterine bleeding	Cutaneous	Known	Metastatic	Endometrium	None identified at diagnosis; cutaneous, pulmonary, and brain metastases developed during follow-up	Total hysterectomy with bilateral salpingo-oophorectomy, followed by chemotherapy	Died 5 months later with metastatic disease to the skin, lungs, and brain
17 (17)	Korolev et al., 2006	67	Abnormal uterine bleeding	Cutaneous	Known	Metastatic	Endometrium	None	Hysterectomy with bilateral adnexectomy	Alive with no evidence of metastasis at follow up after 7 months
18 (18)	McGettigan et al., 2006	54	Postmenopausal bleeding	Cutaneous	Known	Metastatic	Endometrium; polypoid lesion	Brain, lungs, left axillary lymph node, multiple local recurrences on the left arm	Partial finger amputation, axillary lymph node dissection, prior treatment for brain and lung metastases, hysteroscopic polypectomy	Died 4 months later from neurological complications of brain metastases
19 (19)	Coutts et al., 2006	83	Postmenopausal bleeding	Choroid/uvea	Known	Metastatic	Endometrial cavity	Widespread metastatic disease involving the liver, lungs, heart, lymph nodes, bone, kidney, pancreas, spleen, adrenal glands, thyroid, skin, mesentery, bladder, and right ovarian fibroma	Palliative care; patient was not fit for chemotherapy	Died 2 months after presentation; cause of death was cardiac tamponade due to malignant pericardial effusion from cardiac metastasis
20 (20)	Fambrini et al., 2008	52	Irregular uterine bleeding	Cutaneous	Known	Metastatic	Endometrium; polypoid lesion	None	Total hysterectomy with bilateral salpingo-oophorectomy and bilateral pelvic lymphadenec 22tomy, followed by	Disease-free at 6-month follow-up

									3 cycles of dacarbazine	
21 (21)	Moreno Domingo et al., 2008	75	Vaginal bleeding	Primary vaginal malignant melanoma	Known	Metastatic	Endometrium	None initially, later disseminated disease	Wide vaginal excision + bilateral inguinal lymphadenectomy + modified radical hysterectomy + pelvic lymph node dissection	Local pelvic recurrence at 5 montbs followed by widespread dissemination
22 (22)	Wong et al., 2012	54	Uterine lesion detected during routine gynecological examination	Cutaneous	Known	Metastatic	Endometrium; polypoid lesion	Liver, brain, left inguinal lymph node, left breast, back	Chemotherapy/immunotherapy, stereotactic radiotherapy, lymph node dissection, hysteroscopic resection, pelvic radiotherapy	No recurrence at 8 months follow up.
23 (23)	Little et al., 2018	59	Abnormal uterine bleeding	Brain	Known	Metastatic	Endometrium; polypoid lesion	None identified at diagnosis	Total abdominal hysterectomy with bilateral salpingo-oophorectomy, followed by dabrafenib and trametinib	Pelvic metastatic recurrence 2 months after surgery
24 (24)	Song et al., 2019	67	Abnormal uterine bleeding	Cutaneous	Known	Metastatic	Endometrium; polypoid mass at uterine fundus	Left rib and adjacent soft tissue	Laparoscopic-assisted vaginal hysterectomy, bilateral salpingo-oophorectomy, pelvic and para-aortic lymph node dissection.	Uneventful postoperative recovery.
25 (25)	Ulmer et al., 2021	81	Postmenopausal bleeding	Cutaneous	Known	Metastatic	Endometrium; sessile polyp	Widespread metastatic disease, including CNS and lumbar spine metastases	Hysteroscopic polypectomy; gamma knife surgery for intracranial metastases, radiotherapy to lumbar spine lesions, and ipilimumab plus nivolumab	Died less than 7 weeks after diagnosis

									were recommended	
26 (26)	Fernandes et al., 2022	47	Urinary hesitancy, urgency, dysuria and gross hematuria; passage of a dark vaginal mass	Cutaneous	Known	Metastatic	Endometrium	Urinary bladder, left inguinal lymph nodes, spleen, lungs, and brain	Wide local excision and sentinel lymph node excision, followed by adjuvant nivolumab	Not reported
27 (27)	Shi et al., 2024	73	Postmenopausal bleeding	Endometrium	Primary	Primary	Endometrium	Pelvic and para-aortic lymph nodes; gastric fundus	Radical hysterectomy with BSO, pelvic and para-aortic lymph node dissection, followed by 4 cycles of temozolomide plus cisplatin; subsequently dabrafenib and trametinib	Alive and under regular follow-up on targeted therapy at publication
28 (28)	Subramani et al., 2025	55	Postmenopausal vaginal bleeding	Cutaneous	Known	Metastatic	Endometrium	Subcutaneous right leg metastasis; abdominal, retroperitoneal, pelvic and popliteal lymph nodes; later chest and abdominal wall deposits, ileal involvement and perirectal lymph nodes	Temozolomide for 6 months	Died from disease-related complications

*It was stated that “throughout the myometrium there was transmural infiltration…involving virtually the entire uterus”

† Only abstract could be retrieved

References

1. Weber J. [Uterine metastasis of a malignant cutaneous melanoma]. Zentralblatt Allg Pathol U Pathol Anat. 1. januar 1971;114(5):551–3. PubMed PMID: 5291084.

2. Casey JH, Shapiro RF. Metastatic melanoma presenting as primary uterine neoplasm: A case report. Cancer. 1974;33(3):729–31. doi:10.1002/1097-0142(197403)33:3<729::AID-CNCR2820330318>3.0.CO;2-N

3. Takeda M, Diamond SM, DeMarco M, Quinn DM. Cytologic diagnosis of malignant melanoma metastatic to the endometrium. Acta Cytol. 1978;22(6):503–6.

4. Wood C. Metastatic melanoma simulating a primary endometrial tumor. Am J Obstet Gynecol. august 1978;131(7):820–1. doi:10.1016/0002-9378(78)90259-4

5. Scholz P, Eckert N. [Metastases in abrasive material of malignant melanoma in skin (author's transl)]. Zentralbl Gynakol. 1980;102(17):1009–11.

6. Bauer RD, McCoy CP, Roberts DK, Fritz G. Malignant melanoma metastatic to the endometrium. Obstet Gynecol. 1984;63(2):264–8.

7. Nagy P, Csaba I, Kádas I. Malignant melanoma metastatic to the endometrium. Cytologic findings in a direct endometrial sample. Acta Cytol. 1990;34(3):382–4.

8. Pommerenke F, Tessmann D. [Endometrium metastasis of a malignant melanoma of the skin]. Zentralbl Gynakol. 1989;111(5):324–8.

9. Glaubitz M, Kauffels W. [Malignant melanoma in gynecology]. Zentralbl Gynakol. 1992;114 2:59–67.

10. Luxman D, Jossiphov J, Cohen JR, Wolf Y, David MP. Uterine metastasis from vulvar malignant melanoma. A case report. J Reprod Med. 1997;42(4):244–6.

11. Suenaga Y, Katabuchi H, Okamura H, Kageshita T, Ono T. Increased Serum Levels of 5-S-Cysteinyldopa and Intercellular Adhesion Molecule-1 in a Patient with a Uterine Amelanotic Metastasis from a Primary Vaginal Malignant Melanoma. Gynecol Oncol. 1999;72(1):107–10. doi:10.1006/gyno.1998.5176

12. Heinig J, August C, Beckmann V, Konieczny A. Endometrial metastasis of cutaneous melanoma - A case-report bearing diagnostic difficulties -. Zentralblatt Für Gynäkol. 2001;123(9):534–5. doi:10.1055/s-2001-18227

13. Berker B, Sertcelik A, Kaygusuz G, Unlu C, Ortac F. Abnormal uterine bleeding as a presenting sign of metastasis to the endometrium in a patient with a history of cutaneous malignant melanoma. Gynecol Oncol. 2004;93(1):252–6. doi:10.1016/j.ygyno.2004.01.012

14. Varras M, Kassanos D, Tzaida O, Bournas N, Panagiotopoulos N, Chrelias C, m.fl. Ultrasonographic evaluation of a uterine malignant melanoma presenting as a large pelvic mass. J Clin Ultrasound. 2005;33(8):424–6. doi:10.1002/jcu.20143

15. Di Tommaso L, Rahal D, Bresciani G, Roncalli M. Cutaneous melanoma metastatic to uterine adenomyoma: report of a case. Int J Surg Pathol. 2005;13(2):223–5. doi:10.1177/106689690501300217

16. Giordano G, Gnetti L, Ricci R, Merisio C, Melpignano M. Metastatic extragenital neoplasms to the uterus: a clinicopathologic study of four cases. Int J Gynecol Cancer. januar 2006;16:433–8. doi:10.1136/ijgc-00009577-200602001-00081

17. Korolev AA, Golubev OA. [A rare variant of malignant melanoma progression]. Arkh Patol. 2006;68(4):42–42.

18. McGettigan CP, Armstrong G, Saleh N, Slade RJ. Malignant melanoma of the uterus diagnosed at hysteroscopy. J Obstet Gynaecol. januar 2006;26(4):383–5. doi:10.1080/01443610600636004

19. Coutts MA, Borthwick NJ, Hungerford JL, Cree IA. Post-menopausal bleeding: a rare presentation of metastatic uveal melanoma. Pathol Oncol Res. 2006;12(3):184–7. doi:10.1007/BF02893367

20. Fambrini M, Andersson KL, Buccoliero AM, Pieralli A, Livi L, Marchionni M. Late solitary metastasis of cutaneous malignant melanoma presenting as abnormal uterine bleeding. J Obstet Gynaecol Res. 2008;34(4pt2):731–4. doi:10.1111/j.1447-0756.2008.00917.x

21. Moreno Domingo J, Burgos Bretones J, Díez Lázaro S, Ocerin Bengoa I, Vidaurrázaga Olivares N, Díez García J, m.fl. Metástasis endometrial de melanoma maligno. Prog Obstet Ginecol. oktober 2008;51(10):628–34. doi:10.1016/S0304-5013(08)73326-0

22. Wong VV, Kalu G. Metastatic endometrial malignant melanoma. J Obstet Gynaecol. oktober 2012;32(7):714–5. doi:10.3109/01443615.2012.706664

23. Little J, Braniff K. Endometrial metastasis of primary malignant melanoma of the brain. BMJ Case Rep. 2018;2018:bcr-224435. doi:10.1136/bcr-2018-224435

24. Song HS, Kim MH, Lee MY, Jeon SJ, Kim HS, Kim YA, m.fl. Cutaneous malignant melanoma metastatic to endometrium manifesting abnormal uterine bleeding in postmenopausal woman: a case report. J Obstet Gynaecol. 2019;39(8):1184–5. doi:10.1080/01443615.2019.1588236

25. Ulmer K, Powers J, Alrwashdeh A, Hardy-Fairbanks A. Metastatic malignant melanoma in an endometrial polyp. Dermatol Online J. 2021;27(4). doi:10.5070/D3274053161

26. Fernandes MC, Shoushtari A, Leitao Jr MM, Chui MH, Al-Ahmadie H, Petkovska I. Metastatic melanoma concurrent to the urinary bladder and endometrium: Case report. J Med Imaging Radiat Oncol. 2022;66(5):643–6. doi:10.1111/1754-9485.13356

27. Shi X, Shi J, Li Y, Li X, Hu X, Gao H. Primary Malignant Melanoma of the Endometrium: A Case Report. Case Rep Oncol. 2024;17(1):370–6. doi:10.1159/000535635

28. Subramani R, Selvam V, Singh V, Abhilash S. Navigating the Rare: 18F FDG PET/CT Reveals Uterine Metastasis from Cutaneous Melanoma. Indian J Nucl Med. september 2025;40(5):309–11. doi:10.4103/ijnm.ijnm_73_25
